# Pathogen-Derived Carbohydrate Recognition in Molluscs Immune Defense

**DOI:** 10.3390/ijms19030721

**Published:** 2018-03-03

**Authors:** Weilin Wang, Xiaorui Song, Lingling Wang, Linsheng Song

**Affiliations:** 1 Liaoning Key Laboratory of Marine Animal Immunology, Dalian Ocean University, Dalian 116023, China; wangweilin@dlou.edu.cn (W.W.); songxiaorui@dlou.edu.cn (X.S.); wanglingling@dlou.edu.cn (L.W.); 2 Functional Laboratory of Marine Fisheries Science and Food Production Processes, Qingdao National Laboratory for Marine Science and Technology, Qingdao 266235, China; 3 Liaoning Key Laboratory of Marine Animal Immunology and Disease Control, Dalian Ocean University, Dalian 116023, China

**Keywords:** carbohydrate complex, pattern recognition receptor, molluscs, immune recognition, innate immunity

## Abstract

Self-nonself discrimination is a common theme for all of the organisms in different evolutionary branches, which is also the most fundamental step for host immune protection. Plenty of pattern recognition receptors (PRRs) with great diversity have been identified from different organisms to recognize various pathogen-associated molecular patterns (PAMPs) in the last two decades, depicting a complicated scene of host-pathogen interaction. However, the detailed mechanism of the complicate PAMPs–PRRs interactions at the contacting interface between pathogens and hosts is still not well understood. All of the cells are coated by glycosylation complex and thick carbohydrates layer. The different polysaccharides in extracellular matrix of pathogen-host are important for nonself recognition of most organisms. Coincidentally, massive expansion of PRRs, majority of which contain recognition domains of Ig, leucine-rich repeat (LRR), C-type lectin (CTL), C1q and scavenger receptor (SR), have been annotated and identified in invertebrates by screening the available genomic sequence. The phylum Mollusca is one of the largest groups in the animal kingdom with abundant biodiversity providing plenty of solutions about pathogen recognition and immune protection, which might offer a suitable model to figure out the common rules of immune recognition mechanism. The present review summarizes the diverse PRRs and common elements of various PAMPs, especially focusing on the structural and functional characteristics of canonical carbohydrate recognition proteins and some novel proteins functioning in molluscan immune defense system, with the objective to provide new ideas about the immune recognition mechanisms.

## 1. Introduction

All of the organisms, including prokaryote, plant, and animal are facing potentially life-threatening infections by a variety of microbial pathogens. The survival of the host depends on its ability to recognize infectious microbes and induce appropriate defense responses [1,2]. The innate immune recognition is a fundamental and core reaction to sense invaders and trigger the following immune protection. Since the discovery of Toll like receptors (TLRs) in 1990s, the mechanism of immune recognition between hosts and pathogens has been gradually uncovered, with the pattern recognition mechanism proposed in 2002 as a notable milestone [3]. So far, various pattern recognition receptors (PRRs) have been reported to induce immune defense in plants, invertebrates, and vertebrates by recognizing conserved structures of different pathogens, called pathogen-associated molecular patterns (PAMPs).

**Figure 1:**
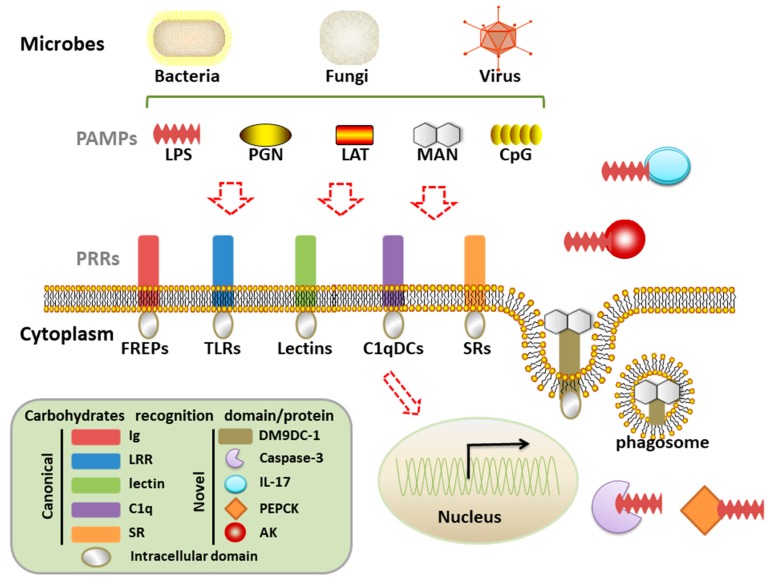
Canonical and novel carbohydrate recognition proteins functioning in molluscan immune defense system. The canonical pattern recognition receptors (PRRs) are mainly composed of five dominant recognition domains, e.g., Ig domain, leucine-rich repeat (LRR) domain, lectin domain, C1q domain, and scavenger receptor (SR) domain. By recognizing (red dotted arrows) the conserved pathogen-associated molecular patterns (PAMPs) of bacteria, fungi and viruses, these PRRs could activate cellular or humoral immune defense and protect host from infection. Except for the canonical PRRs, some new proteins were identified with pathogen derived carbohydrate recognition activity in molluscs, including novel DM9 domain containing protein 1 (DM9DC-1) and some other well-known proteins whose PRR function was discovered recently. (Lipopolysaccharide, LPS; Peptidoglycan, PGN; Lipoteichoic acids, LTA; Mannan, MAN; Fibrinogen-related proteins, FREPs; Toll like receptors, TLRs; C1q domain containing proteins, C1qDCs; Scavenger receptors, SRs; Phosphoenolpyruvate carboxykinase, PEPCK; Arginine kinase, AK; black arrow, gene expression).

In the long-term evolution, the immune protection first starts with non-professional immune cells in plant, cnidarians, and nematode, and the epithelial cells are able to mediate innate immune responses [4]. The true cavity is developed from the endomesoderm of triploblastica and is filled with body fluid, which is concomitant with the presence of open circulatory system and specialized immunocytes (hemocytes) in ancient mollusc [5,6]. The molluscan immune system could be inferred as the most ancient prototype of highly specialized immune system of advanced vertebrates. Moreover, the phylum Mollusca is one of the largest groups in the animal kingdom with abundant biodiversity, which provides plenty of natural solutions about pathogens defense [7]. Accordingly, the study of molluscan immune recognition will be of great reference for understanding the common codex behind nonself recognition. In this concern, the present review introduces the common elements of various PAMPs and diverse PRRs, with the emphasis on the canonical and novel carbohydrate recognition proteins in molluscs (Figure 1).

## 2. Carbohydrate as a Main Element of PAMPs in Microbes

There are four major classes of biological macromolecules that are required for all the living organisms, including carbohydrates, proteins, nucleic acids, and lipids. They are composed of different types of subunit (sugars, amino acids, nucleotides, and fatty acids) and exhibit different profile in component, structure, and distribution. For example, the polysaccharides in the extracellular matrix (ECM) of algae, plants, bacteria, fungi and animals are different, such as cellulose in plants, peptidoglycan (PGN) in bacteria, chitin and glucans in fungi, and sulfated polysaccharides in animals [8]. The four individual macromolecules or their complex constitute the main elements of PAMPs in diverse pathogens. For example, lipopolysaccharide (LPS), peptidoglycan (PGN), lipoteichoic acids (LTA), and cell-wall lipoproteins are the main bacterial PAMPs. β-glucan (GLU) and mannan (MAN), the typical components on cell walls of fungi, are the fungal PAMPs [9]. Ribonucleic acid or deoxyribonucleic acid, which are often mimicked by poly I:C or CpG, are considered as the main targets for the immune system to recognize virus. These PAMPs show a common feature in that their chemical bases are all derived from one of the four macromolecules or two complexes (Table 1).

Carbohydrates (glycans), as the most stable organic macromolecule with complex structure, have long been known to play fundamental roles in antigen formation and innate immune recognition [10,11]. Glycosylation is essential for almost all of the organisms, including fungi, yeasts, plants, insects, fishes, birds, and mammals, and most of their secreted and cell-surface proteins are glycosylated [12]. Meanwhile, all of the cells are coated with thick layers of complex carbohydrates, known as the glycocalyx, in which the glycan components are present in many different glycoforms, such as glycoproteins, proteoglycans, and glycolipids [13]. These glyco-complexes either secreted or located on the cell surface are the main molecular components of pathogen-host interaction, and essential for both pathogen invading and host innate recognition [14]. For example, LPS is the common component on the cell surface of Gram-negative bacteria, which can be recognized by TLR, C-type lectin (CTL), and scavenger receptor of host innate immunity as PAMP to recognize the invaded bacteria [3]. The other carbohydrates as main elements of PAMPs in microbes are also the ligands of various PRRs of host immunity to recognize the pathogens.

## 3. Main Recognition Domains of PRRs in Various Organisms

With the long evolution, all organisms have developed an effective immune recognition system, in which several families of germline-encoded PRRs can detect the conserved molecular patterns of microbes. Recently, a picture of the evolutionarily conserved sensing system has emerged by comparing the structural homologies of these pathogen sensors across species [15]. Most PRR families with evolutionarily conserved structures execute function by forming common motif and binding sites. For example, toll like receptors (TLRs) in simple nematode, diverse molluscs, and complex mammals recognize various PAMPs through the conserved LRR domains.

In terms of structure, almost all of the PRRs contain specific domains classified into different types. Immunoglobulin (Ig) domain, leucine-rich repeat (LRR) domain, C-type lectin (CTL) domain, C1q domain, and scavenger receptor (SR) cysteine-rich domain are identified as the most prominent motifs, which are also rearranged with various combinations to build immune recognition rhyme [15]. For example, fibrinogen-related proteins (FREPs) are composed of tandem Ig domains, and TLRs are composed of varied number of LRR domains (Table 2). The recognition characters of each domain endow a PRR with functions to recognize and discriminate specific ligands.

Recently, species-specific expansion of recognition domains has been significantly characterized in plants and invertebrates by screening the available genomic sequence. There is a big family of LRR containing recognition proteins reported in plant *Arabidopsis* [16], and 187 FREPs with Ig domains annotated in sea anemone *Nematostella vectensis* [17]. In oyster *Crassostrea gigas*, there is significant expansion of C1q, CTL, and Ig recognition domains, and 321 C1qDCs, 266 CTLs, and 190 FREPs [17] are annotated in its genome. The massive increases of LRR (222 TLRs and 203 NLRs), SR (218 SRs) and CTL (283 CTLs) recognition domains are also reported in sea urchin *Strongylocentrotus purpuratus* [17]. These recognition domain containing proteins play an indispensable role in the first step of immune defense, and the research progresses have greatly expanded our understanding of pattern recognition mechanisms. Some of their detailed characteristics and functions in carbohydrate recognition are discussed below. 

## 4. Canonical Carbohydrates Recognition Protein in Molluscs

The sophisticated repertoire of PRRs in molluscs could be mainly classified into several families, depending on their recognition domains, such as Ig domain containing PRRs (FREPs, IgSF proteins), LRR domain containing PRRs (TLRs and LRRs), lectin domain containing PRRs (CTL, Galectin), C1q domain containing PRRs (C1qDC), SR domain containing PRRs (SRs), and other domain containing PRRs (PGRPs, GNBPs) (Table 2). These molluscan PRRs are canonical protein to recognize carbohydrates-complex of invaders.

### 4.1. Ig Domain Containing Pattern Recognition Receptors

Ig domain is an evolutionarily ancient domain, and it can tandemly build a functional protein or assemble complexes with other domains. An individual Ig domain is approximately 100 amino acids in length and forms an “Ig-fold” structure, consisting of two anti-parallel β-sheets packed face to face [18]. Four sets of Ig domains have been described as variable-like domains (V), constant-like domains (C1 and C2), and intermediate domains (I) [18]. So far, varied numbers of Ig superfamily (IgSF) have been identified in different phylogenetically organisms, including FREPs in molluscs [19,20], Dscams in arthropods, and Variable region-containing chitin-binding proteins (VCBPs) in echinoderm [21]. Though lacking Ig dependent adaptive immunity, molluscs might be endowed with enhanced innate immunity by the recognition, adhesion, and opsonic roles of these Ig domain containing proteins.

#### 4.1.1. Fibrinogen-Related Proteins (FREPs)

FREP is a kind of Ig and fibrinogen-like (FBG) domain containing protein with high levels of sequence diversity, and it is involved in innate defense responses of invertebrates [22]. FREPs have been recently identified in a variety of mollusc, such as oyster *C. gigas* [19,23], snail *Biomphalaria glabrata* [24,25], and marine opisthobranch *Aplysia californica* [26]. There are about 190 predicted FREP genes with more than 200 FBG domains and 70 FBG-encoding genes identified in the genome of *C. gigas* [23] and *Lottia gigantea* [27], respectively. The large amount of FREPs suggests that they have considerable functional significance in the molluscan immune system. In addition, just like Dscam molecules in arthropods, the FREPs reported in molluscs are of abundant sequential polymorphism in their Ig domains. In *B. glabrata*, a surprising diversity of FREP genes were generated through point mutation and recombination [28], and the finding of alternatively spliced forms of FREPs transcripts and retrosequences suggested that their diversification occurred at both genomic and transcriptional levels [24,29]. But, the detailed mechanisms of FREPs polymorphism still need further studies.

Molluscan FREPs have been reported to be involved in immune response as an important kind of PRRs, mainly with binding and opsonic activities [30]. The mRNA expression of molluscan FREPs can be induced by PAMP stimulations, and FREP proteins exhibit varied binding activities towards diverse microbes. The mRNA expression level of FREPs in mussel *M. galloprovincialis* was significantly up-regulated after LPS, LTA, poly I:C, and zymosan stimulations [31]. *Ai*FREP-1 from scallop *Argopecten irradians* could agglutinate Gram-negative bacteria *E*. *coli* JM109, *V*. *anguillarum* and Gram-positive bacteria *M*. *luteus* in the presence of calcium ions [32]. *Ai*FREP-2 could bind different PAMP ligands including LPS, PGN and GLU, and various microbes, including Gram-negative bacteria (*V*. *anguillarum*), Gram-positive bacteria (*Staphylococcus aureus*), and fungi (*Pichia pastoris* and *Yarrowia lipolytica*) [33]. FREPs from *B. glabrata* are also able to bind trematode *Echinostoma paraensei* sporocysts, as well as a variety of microbes (Gram-positive and Gram-negative bacteria and yeast) with certain specificity with respect to pathogen type [29]. FREP4 of 65–75-kDa could bind to sporocysts and secretory/excretory products of *E. paraensei*, whereas FREPs with higher molecular-weight bound to Gram-positive, Gram-negative bacteria, and yeast [29]. Except for the binding activity, FREPs are also found to promote cellular immunity. The purified FREPs from serum of mussel *M. galloprovincialis* were reported to promote the phagocytosis of fluorescent beads [31].

However, the mechanisms of binding specificity of molluscan FREP and the polymorphism of Ig domain still need further investigation. The identification of more FREPs and their polymorphisms, and the comparison of structure and function will further clarify the taxonomic diversification of FREPs and their underlying recombination mechanisms in molluscs [20], providing clues to understanding the relationship between the diversification of FREP and specific immunity.

#### 4.1.2. Siglec and JAM-A Protein

Except for FREPs, several Ig domain only proteins have also been identified as vital PRRs in the mollusc immune system. Siglec is a member of IgSF, which involves in the host-pathogen recognition, cell–cell interactions and subsequent signaling pathways in the immune and nervous systems in vertebrates. The orthologs of mammalian Siglec are composed of one N-terminal V-set Ig domain, C2-set Ig domains, and cytosolic immunoreceptor tyrosine-based inhibitory motifs (ITIMs) [34,35]. The key arginine (Arg) residues in N terminal V-set Ig domain endow Siglecs with an ideal platform for sialic acid binding through a salt bridge [35]. Recently, a homolog of Siglec was identified in oyster *C. gigas* (*Cg*Siglec-1) with two I-set Ig domains [36]. The recombinant *Cg*Siglec-1 protein (r*Cg*Siglec-1) could bind poly sialic acid (pSIAS), LPS, and PGN in a dose-dependent manner, which was different from other Siglecs characterized by binding ligands only in a sialic acid-dependent manner [36]. The significant difference might attribute to the N-terminal I-set Ig domain instead of the canonical V-set. The blockade of *Cg*Siglec-1 by specific polyclonal antibodies enhanced the LPS-induced cell apoptosis and phagocytosis towards *V. splendidus*. The broader PAMP binding spectrum and the induction of cellular immunity might endow *Cg*Siglec-1 with important roles in the immune response of oyster against a variety of foreign invaders [36]. Junctional adhesion molecule (JAM), another example of IgSF with a couple of immunoglobulin domains, can act as a regulator in homeostasis and inflammation in vertebrates [37]. A structural homolog of JAM-A (designated *Cg*JAM-A-L) was identified from oyster *C. gigas* with typical JAM-A domain, which functioned as an adhesion molecule [38]. The recombinant *Cg*JAM-A-L could bind to LPS, PGN, LTA, MAN, GLU, and poly I:C, as well as various microorganisms including *M*. *luteus*, *S. aureus*, *E*. *coli*, *V. anguillaru*m, *V. splendidus*, *P. pastoris,* and *Y. lipolytica*. The phagocytic rates of oyster hemocytes towards Gram-negative bacteria *V. anguillarum* and yeast *P. pastoris* were significantly enhanced after the incubation of r*Cg*JAM-A-L [38]. These results collectively indicated that the Ig containing proteins in mollusc could function as recognition molecules and opsonins in the immune defense against invading pathogen. As the most primitive specie with homolog of Ig, the Ig containing proteins (such as *Cg*Siglec and *Cg*JAM-A) in mollusc would provide useful clews for the evolutionary study of Ig domains as well as the adaptive immunity.

### 4.2. LRR Domain Containing Pattern Recognition Receptors

LRR domain is a most widespread protein domain, which has a conserved core consensus of L-x-x-L-x-L-x-x-N to form a β-strand followed by a more variable sequence [39]. PDB structures for LRR-containing proteins show the LRR domains in an arc or horseshoe shape [39]. The function of many LRR domains is to provide a structural framework for protein–protein interactions (PPIs) [39]. More than 60,000 LRR-containing proteins have been identified in viruses, bacteria, archaea, fungi, plants, and animals [40]. They are especially enriched in plants [16], invertebrates (e.g., sea urchin) [41], and Cephalochordata (e.g., amphioxus) [42], which lack adaptive immunity. In plants, only two kinds of PRRs have been reported, and all of them are composed of LRR domains [16]. In animals, LRR-containing proteins, such as Toll-like receptors (TLRs) and NOD-like receptors (NLRs), could recognize the molecular determinants from a structurally diverse collection of bacterial, fungal, viral and parasite-derived components via the LRR domain [43]. It is worth noting that the selectively assembling of LRR segments in variable lymphocyte receptor gene of jawless vertebrates generates structural diversity for antigen recognition, which builds a new alternative adaptive immune system [44].

The LRR-containing proteins could be divided into two groups, proteins containing LRR motif and other motifs or domains, and proteins merely containing LRR motif termed LRR-only proteins [45]. The TLRs are typical representatives for the former group, consisting of a solenoid-like LRRNT (LRR N-terminal)-LRR-LRRCT (LRR C-terminal) ectodomain for ligand recognition and a cytoplasmic TIR domain for signal transduction [46]. As a family of conserved PRRs, TLRs are widely distributed in nearly all animal phyla, and they are well studied in mollusc. So far, a series number of TLRs have been identified in molluscs and an expanded set of 83 TLR genes are annotated in the oyster genome, which can be divided into five groups based on the patterns of sequence relatedness [17]. Among them, sP-type (short protostome-like without LRRCT-LRRNT ectodomains) TLRs are the most greatly expanded PRRs in oyster [39], which are similar with equally extensive expanded P-type TLRs (protostome-like with LRRCT-LRRNT ectodomains) in *Drosophila* [47] and V-type (vertebrate-type) TLRs in the sea urchin [41], suggesting that species-specific TLR gene expansion is a frequent, independent occurrence in metazoan phylogeny. Moreover, the expanded oyster TLRs displayed a different expression profile induced by single or different combinations of bacteria/virus stimulation [17]. For example, the expression of TLR *Cg*26493-D2 elevated after challenges with LPS and all *Vibrio* strains, while *Cg*13671 was only induced by *V. anguillarum*. About ten bivalve TLRs have been identified to participate in immune recognition of various microbes and PAMPs. A newly identified non-phagocytic receptor *Cg*TLR-6 from *C. gigas* exhibited affinity to LPS and PGN, and broader recognition spectrum for bacteria and fungi [48]. Astonishingly, *Cg*TLR1–4 from *C. gigas* lack the activities to recognize specific PAMPs, but they can activate NF-κB responsive reporter in HEK293 cells with a dose-dependent manner [49], which supports the functional differentiation of these primordial TLRs in molluscs. Moreover, three TLRs (*Hc*Toll1, *Hc*Toll-2, and *Hc*Toll3) from the pearl mussel *H. cumingii* could respond to bacterial and viral challenge and regulate the expression of antimicrobial peptides (AMPs) [50,51,52]. A rather canonical MyD88-dependent TLR pathway was also demonstrated in scallop and this pathway was involved in immune signaling to activate downstream anti-oxidant, anti-bacteria reaction, and apoptosis [53]. Recently, 29 TLR signaling pathway components were identified in Zhikong scallop *Chlamys farreri*, including SARM (ORF08051) and TRAM/TICAM2 (ORF08726), indicating the possibility of MyD88-independent TLR signaling pathway in mollusc [54]. All of the evidences suggested that the ancient molluscan TLRs could recognize broader pathogens and related PAMPs, and mediate the downstream anti-bacteria reaction in a MyD88-dependent and MyD88-independent dependent pathway. 

The versatile functions of proteins merely containing LRR motif (LRR-only proteins, LRRop) in immune response have also been recently reported in scallop *C. farreri*. Six *Cf*LRRops (*Cf*LRRop 1-6) containing varied number (1–4) of LRR domains have been identified [40,55,56] with different mRNA expression profiles and PAMP binding activities. The mRNA expression of *Cf*LRRop-1, -2, and -3 in hemocytes could be induced by the stimulations of LPS, PGN, GLU, and poly I:C, while the mRNA transcripts of *Cf*LRRop-4, -5, and -6 in hemocytes could respond to the stimulations of different microbes, including *V*. *anguillarum*, *M*. *luteus,* and *P*. *pastoris*. The recombinant *Cf*LRRop-1, -3, -4, -5, -6 proteins, but not r*Cf*LRRop-2 protein, could directly bind LPS, PGN, GLU, and poly I:C with varied affinity, indicating LRR-only proteins represented a new type of PRR in bivalve with similar recognition characters as TLR [40,55,56]. Additionally, r*Cf*LRRop-1 and -2, but not r*Cf*LRRop-3, could induce the release of TNF-α in primary cultured scallop hemocytes. r*Cf*LRRop-4, -5, and -6 could induce varied immune effectors, including tumor necrosis factor α, superoxide dismutase, catalase, and lysozyme. The versatile *Cf*LRRops could function in immune response not only as PRRs, but also as an immune effector or pro-inflammatory factor, suggesting a functional differentiation among the diverse *Cf*LRRops [40,55,56]. These preliminary results, together with future characterization of the large variety of unexplored LRRops in molluscs, will offer valuable information for understanding the comprehensive mechanism of immune recognition [40,55,56].

### 4.3. Lectin Domain Containing Pattern Recognition Receptors

Lectins are the most classical carbohydrate-binding proteins to bind the glycans of glycoproteins and glycolipids with high affinity. They play crucial roles in the innate immune responses of invertebrates by recognizing and eliminating pathogens [57]. Based on the structure, animal lectins have been classified into at least 13 lectin families (Available online: www.imperial.ac.uk/research/animallectins/), and at least seven groups of lectins have been identified in mollusc, including C-type, P-type, F-type, I-type, galectins, ficolins, and chitinase-like lectins [58,59]. Two extracellular lectins including C-type lectins (CTLs) and galectins are discussed in this circumstance with the emphases on their conserved domains and functions, such as cell adhesion, cell signaling, glycoprotein clearance, and pathogen recognition in molluscs. 

#### 4.3.1. C-Type Lectins (CTLs)

CTLs are structurally characterized by double-loop composed of two highly conserved disulfide bridges located at the bases of the loops, and they can recognize and bind to terminal sugars on glycoproteins and glycolipids in a calcium-dependent manner [58,60]. Generally, CTLs contain at least one carbohydrate-recognition domain (CRD) with about 120 residues. Presently, a more general term “C-lectin domain containing proteins” (CTLDCs) has been introduced to define the proteins with one or more CTLDs, regardless of carbohydrate or calcium binding ability [58]. CTLs are abundant in molluscs, and an expanded set of 266 CTLDCs were annotated in the genome of oyster *C. gigas* [17]. More than 30 CTLs have been identified in molluscs, one from *Venerupis philippinarum* (*Vp*CTL) [61], two from abalone *Haliotis discus hannai* (CLHd and *Hdh*CTL1) [62,63], *Hyriopsis cumingii* (*Hc*perlucin and *Hc*Lec4) [64,65], and Manila clams *Ruditapes philippinarum* (MCL-3 and MCL-4) [66,67,68], respectively, six from *C. farreri* (*Cf*Lec-1-*Cf*Lec-4, *Cf*Lec-4b, *Cf*Lec-5) [7], seven from oyster *C. gigas* (*Cg*CLec-1-*Cg*CLec-7) [69], and ten from bay scallop *A. irradians* (*Ai*CTL-1-*Ai*CTL-10) [7]. Accumulating evidences have favored that, CTLs in molluscs exhibit high diversity of structure and functions.

The diversity of CRD structure is mainly embodied in the number of CRD as well as variety of Ca^2+^-binding site 2 in CRDs. Most molluscan CTLs contain single CRD, while there are three or four CRDs in *Cf*Lec-3 and *Cf*Lec-4 from *C. farreri* [70,71] and *Ai*CTL-9 from *A. irradians* [72], respectively. The architecture and phylogenic analysis revealed that multi-domain CRDs in different lineages did not arise from a common multi-domain progenitor, and these proteins served distinct functions in different animal lineages [70]. The CRD contains a double-loop structure, and the second loop (also called long loop region) is involved in Ca^2+^-dependent carbohydrate binding. There are four Ca^2+^-binding sites in this structure, among which the site 2 is known to be involved in the carbohydrate binding [58,73,74]. For the Ca^2+^-binding sites 2, there are two conserved motifs determining the CRD binding ability. In vertebrates, the first motif is always EPN (Glu-Pro-Asn) or QPD (Gln-Pro-Asp), and the second one always WND (Trp-Asn-Asp). While in invertebrates, much more diversities are reported, and there are at least seven types in the first motif and more than ten types in the second motif presented in different molluscan CTLs [59]. The first motif is considered as the key switch in the specificity of binding with carbohydrate, and the second one increases the binding affinity and specificity [73,75]. However, the information about the function of the second motif in molluscs is still limited, which is urgently needed for us to understand the mechanism of CTLs functioning as a PRR.

Diverse immunological functions of C-type lectins in molluscs have been demonstrated in the past decades. The mRNA transcripts of almost all the CTLs are increased after stimulation with pathogens or PAMPs, implying that they are involved in innate immune response [67,76]. As an important PRR, molluscan CTLs display high affinity to various PAMPs on the surface of pathogens, such as LPS from Gram-negative bacteria, PGN from Gram-positive bacteria, glucan and mannan from fungi, and so on. Especially, the clustering of multiple CRDs in one molecule showed different PAMPs binding spectrum and affinity, and thus endowed multi-CRD CTLs with a broader spectrum and higher affinity to bind PAMPs. For instance, *Cf*Lec-3 from *C. farreri* with three CRDs can bind more PAMPs than *Cf*Lec-1 and *Cf*Lec-2 with single CRD [77]. *Ai*CTL-9, a multi-CRD lectin from scallop *A. irradians,* can bind LPS, PGN, glucan, and mannan [72]. Among the recombinant proteins of four CRDs in *Cf*Lec-4 from *C. farreri* (designated as rCRD1, rCRD2, rCRD3, and rCRD4), rCRD3 and rCRD4 could bind to all four PAMPs tested, while rCRD1 and rCRD2 could only bind to LPS and mannan [78]. It is noteworthy that molluscan CTLs with the same first motif of Ca^2+^-binding site 2 displayed different PAMPs binding spectrums. In *C. farreri*, *Cf*Lec-1 containing the motif EPD could bind LPS, PGN and mannan in vitro, while *Cf*Lec-2 with the same motif could also bind zymosan besides the other three PAMPs [79,80]. Similarly, CRD1 of *Hc*Lec4 in *H. cumingii* with a “LND” motif could bind to LPS and PGN, while CRD3 of *Hc*Lec4 with “LND and EPN” motifs could not bind to LPS and PGN [64]. Besides nonself recognition, molluscan CTLs participate in innate immune responses, including agglutination, hemocyte phagocytosis, and encapsulation, and they even display bactericidal effect. For instance, most of molluscan CTLs exhibit agglutinating activity towards various bacteria and fungi [7]. The purified MCL, MCL-3, and MCL-4 from the plasma of Manila clam *R. philippinarum* not only markedly suppressed the growth of *Alteromonas haloplanktis*, but also significantly enhanced the hemocyte phagocytic ability toward the bacteria [67,81]. Similarly, the recombinant CTLs from *C. farreri* [79,80] and *C. gigas* [82] also inhibited the growth of several bacteria. In *H. cumingii*, the bacteria clearance efficiency and the expression level of AMPs were both increased after *Hc*Lec4 was knocked down, revealing that *Hc*Lec4 exerted its antibacterial effect by regulating the expression of AMPs at the early stage of bacterial infection [64].

A large number of CTLs identified from molluscs shared similar structural features and displayed versatile immunocompetence. Since molluscs lack of antibody mediated adaptive immunity, abundant CTLs with diverse expression profiles and bioactivities might function as nonclonal effectors in the molluscan immune system.

#### 4.3.2. Galectin

Galectins are a family of most conserved and ubiquitous lectin, which is defined by a conserved CRD with a canonical amino acid sequence and affinity primary for β-galactoside [2]. Similar with C type lectins, some galectins have one single CRD, while other galectins contain a clustering of multiple CRDs, such as *Cv*Gal identified from the hemocytes of *C. virginica* [83], *Po*Gal-1 and *Po*Gal-2 from *P. fucata* [84,85], *Ai*Gal-1 and *Ai*Gal-2 from *A. irradian*s [86,87], and MCGal from clam *R. philippinarum* [88]. The clustering of multiple CRDs organization was uniquely found in molluscan galectins, in which different CRDs might share a common ancestor, according to the phylogenetic analysis [83,85]. 

It has been demonstrated that galectin of molluscs can target glycans on the surfaces of bacteria and parasites, and plays a crucial role in innate immune responses [7]. Two galectin in *P. fucata*, *Po*Gal-1 and *Po*Gal-2, were significantly up-regulated after *V. alginolyticus* stimulation, suggesting that *Po*Gals were involved in the immune response against bacteria [84,85]. *Cv*Gal in *C. virginica* facilitated particularly the recognition of a variety of potential microbial pathogens, unicellular algae, and preferentially *Perkinsus* trophozoites [83]. The strong interaction between *Cv*Gal and *P. trophozoites* suggests that *Cv*Gal functions as a hemocyte surface receptor for the parasite and mediates phagocytosis [83]. MCGal exhibited an affinity towards galactose and N acetylgalactosamine and could bind to the surface of *Perkinsus olseni* and agglutinate *V. tapetis* in vitro [88]. *Ai*Gal-2 from *A. irradians* displayed high binding and agglutinating activities toward a variety of microbes, including *V. anguillarum*, *M. luteus*, *E. coli*, *V. fluvialis*, and *Edwardsiella tarda*, and could also recruit hemocytes and promote hemocytes encapsulation. These results suggested that *Ai*Gal2 functioned as a pattern recognition receptor in immune defense and contributed to the nonself recognition and elimination in cellular immune response of bay scallop [87].

#### 4.3.3. Other Lectins

F-type lectins (fucolectins) are fucose-binding proteins, which have also been identified in oyster *Pinctada martensii* (*Pm*F-lectin) and *C. gigas*. The mRNA transcript of *Pm*F-lectin is abundant in hemocytes and gill, and its expression level is dramatically up-regulated after a challenge with *V. alginolyticus*, suggesting that *Pm*F-lectin is involved in the innate immune response [89]. 

The chitinase-like lectins and ficolin-like proteins are also found in molluscs. Two chitinase-like proteins (*Cg*Clp1 and *Cg*Clp2) were identified from *C. gigas*, and their mRNA expressions in hemocytes could be induced by LPS stimulation, strongly suggesting that these two close paralogous genes were involved in oyster immunity [90]. A novel ficolin-like protein (*Ch*FCN) was identified from *C. hongkongensis*. It contained a typical signal peptide and a fibrinogen-related domain at the N- and C-terminus, respectively, but no additional ficolin collagen-like domain. *Ch*FCN mRNA transcripts increased under microbial infections and the recombinant *Ch*FCN protein could bind and agglutinate microorganisms, and enhance the phagocytosis [91].

Recently, some new family lectins were proposed in molluscs. For instance, a glycosylated galactose-binding lectin (MCL) from *M. californianus*, showed antibacterial activities against Gram-positive and Gram-negative bacteria, but didn’t belong to any previously reported lectin family via bioinformatics analysis [92]. A new lectin from *Aplysia dactylomela* eggs (ADEL) was isolated by affinity chromatography with the activities to agglutinate and inhibit biofilm formation of *Staphylococcus aureus*, suggesting that this lectin might be a potential alternative to the conventional use of antimicrobial agents in the treatment of infections caused by *Staphylococcal* biofilms [93].

### 4.4. C1q Domain Containing Pattern Recognition Receptors

C1q domain containing proteins (C1qDCs) are a family of proteins containing a globular head C1q domain (ghC1q) in C-terminus, which recognize a broad range of ligands and trigger a serial of immune responses, such as the classical pathway of complement [94]. There were several C1qDC proteins identified from molluscs, including *M. galloprovincialis* (*Mg*C1q) [95], pearl mussel *Hyriopsis cumingii* (*Hc*C1qDC5) [96], *C. farreri* (*Cf*C1qDC and *Cf*C1qDC-2), *A. irradians* (*Ai*C1qDC-1 and *Ai*C1qDC-2) [7], and *C. gigas* (*Cg*C1qDC-1–4) [7]. All of the molluscan C1qDCs contain a typical gC1q domain with a 10-stranded β-sandwich fold forming a jelly-roll topology, and they are found to exert diverse biological functions in molluscan immunity. For instance, the mRNA expression level of *Mg*C1q in *M. galloprovincialis* hemocytes was drastically increased after the stimulations of Gram-positive bacteria and Gram-negative bacteria [95]. The recombinant *Cf*C1qDC (r*Cf*C1qDC) and *Ai*C1qDC-2 (r*Ai*C1qDC-2) proteins both bound LPS, PGN, β-glucan, and poly I:C, while the latter could also bind mannan and yeast-glucan [97,98]. The broader PAMP binding activities of *Ai*C1qDC-2 contributed to its broader bacterial agglutinating spectrum to fungus *P. pastoris*, Gram-positive bacteria *B. subtilis,* and Gram-negative bacteria *E. coli* and *V. anguillarum* [99]. Moreover, *Cg*C1qDCs in oyster (*Cg*C1qDC-2–4) could enhance the phagocytosis of oyster hemocytes, and the enhancements towards Gram-negative bacteria were significantly higher than that towards Gram-positive bacteria, indicating the important roles of molluscan C1qDC as an opsonin in the clearance of invading microbes [100]. Comparatively, *Cg*C1qDC-3 exhibited higher binding affinity to LPS and stronger opsonization, and its mRNA expression could be up-regulated rapidly and persisted upon the secondary challenge with homologous *Vibrios*. It was proposed to exert efficient functions in the immune response against invading pathogens [100]. Most interestingly, *Cf*C1qDC could interact with human heat-aggregated IgG, which gave a hint for the conservative property of C1qDC proteins to functionally interact with immunoglobulins in evolution [98]. 

An expanded set of 321 and 168 C1qDCs are annotated in oyster [17] and mussel genome [95], which are promising molecules that are involved in immune specificity with functional divergence and provide clues for the knowledge of evolution of the complement system.

### 4.5. SR Domain Containing Pattern Recognition Receptors

SRs represent a large family of endocytic receptors with multifunction to bind and internalize a variety of microbial pathogens, as well as modified or endogenous molecules derived from the host, and contribute to a range of physiological or pathological processes [101]. According to their multi-domain structure, SRs are classified into six different classes (classes A, B, C, D, E, and F) [102]. However, the information about molluscan SRs is extremely limited, and only two SRs have been identified in oyster *P. martensii* and scallop *C. farreri* [103]. The mRNA expression of *Cf*SR was significantly up-regulated by the stimulations of LPS, PGN, and β-glucan. The recombined protein *Cf*SR displayed unique broad ligand-binding properties not only for acetylated low density lipoprotein (Ac-LDL) and dextran sulfate, but also for various PAMPs, such as LPS, PGN, mannan, and zymosan [103]. Moreover, the increasing genomic information indicates that there is an expansion of SRs and about 71 members have been annotated in oyster, and more especially, they have experienced high levels of diversification [17]. The mRNA transcripts of SRs in oyster *C*. *gigas* are highly up-regulated in response to summer mortality syndrome, hypoxia, and bacterial challenge [69], suggesting their significant roles in adaption to complex environment.

### 4.6. Other Domain Containing Pattern Recognition Receptors

#### 4.6.1. Peptidoglycans Recognition Proteins (PGRPs)

PGRPs are a kind of highly conserved PRRs that specifically bind to PGN, a unique cell wall component of all virtual bacteria, but is not present in eukaryotic cells [104]. Nine PGRP genes are predicated in the oyster *C. gigas* genome, and six of them (*Cg*PGRP-S1S, -S1L, -S2, -S3, -S4, and *Cg*PGRP-L) have been characterized [17,69]. A positive Darwinian selection was suggested by the phylogenetic analysis in the *Cg*PGRP family [105]. Molluscan PGRPs have also been identified from the bay scallop *A. irradians* (*Ai*PGRP) [106] and Zhikong scallop *C. farreri* (*Cf*PGRP S1) [107]. The identified molluscan PGRPs are all short type with a conserved amidase-2/PGRP domain in their C-terminus, suggesting that these proteins retain the conserved amidase activity. Although the molecular weight of *Cg*PGRP-L is close to that of long PGRP groups, it is homologous to short PGRPs and possesses an additional goose-type (g-type) lysozyme domain, suggesting that *Cg*PGRP-L may have both binding and lytic functions against the bacterial cell wall [108]. *Cf*PGRP-S1 displayed strong activities to agglutinate Gram-positive bacteria *M. luteus* and *Bacillus subtilis*, while weak activities to agglutinate Gram-negative bacteria *E. coli* [107]. It also functioned as a zinc-dependent amidase to degrade PGN and strongly inhibited the growth of *E. coli* and *S. aureus* in virtue of the conserved Zn^2+^ binding sites in amidase-2/PGRP domain. The recombinant PGRP-S1S (rPGRP-S1S) from oyster *C. gigas* did not show any bactericidal activity, but agglutinated *E. coli* and induced secretion of granular contents by hemocyte degranulation [109]. Although all of the molluscan PGRP genes contain conserved PGRP/amidase domain, the critical residues involved in specific PGN recognition show a certain degree of mutation, which may create a more flexible response to different microbial challenges.

#### 4.6.2. Gram-Negative Bacteria-Binding Proteins (GNBPs)

The GNBP family, including the LPS and β-1,3-glucan binding protein (LGBP), and β-1,3-Glucan binding proteins (βGBP), can bind Gram-negative bacteria, LPS, and GLU. The information about molluscan GNBP is still limited and there are only a few GNBP genes identified from *P. fucata* (poLGBP) [110] and *C. farreri* (CfLGBP) [111], respectively. They both possess a potential polysaccharide-binding motif, a glucanase motif, an LPS-binding site, and a β-1,3-linkage of polysaccharide. Their mRNA expressions were significantly up-regulated after bacteria or LPS stimulation, indicating a possibly important role during bacterial infection [110,111]. The *Cf*LGBP exhibited obvious binding and agglutination activity toward various PAMPs and microorganisms, and the polymorphism of 7679 G/G in *Cf*LGBP enhanced its binding activity to LPS and β-glucan, which was associated with the disease resistance of scallop against *V. anguillarum* [112]. There are two cDNA fragments coding βGBPs (*Cg*βGBP-1 and *Cg*βGBP-2) reported in oyster. Although domain structures of both *Cg*βGBPs are similar to other invertebrate βGBPs, they seem to have evolved with different immunological functions. The recombinant *Cg*βGBP-2 enhanced the phenoloxidase (PO) activity of hemocyte suspensions under the presence of laminarin, but r*Cg*βGBP-1 did not show this enhancement. Because there are integrin recognition sites that areidentified in *Cg*βGBP-1, but not in *Cg*βGBP-2, it is suggested that the hemocyte-related functions of *Cg*βGBP-1 are possibly evolved from integrin [112]. Additionally, a βGBP was purified from the plasma of marine mussel *Perna viridis* with an inherent serine protease activity [113]. It agglutinated baker yeast, bacteria, and erythrocytes, and enhanced pro-PO activity of the plasma. Although the gene sequence and molecular structure are still unknown, molluscan βGBPs are considered to function as recognition molecule for β-1,3-glucan, and also mediation molecule in the immune response such as PO activation in molluscs.

## 5. Novel Carbohydrates Recognition Protein in Molluscs

Except for the canonical carbohydrates recognition proteins, some novel molecules were discovered in molluscs to play roles in PAMPs recognition. Concomitantly, some unconventional immune defense strategies or novel pathopoiesis mechanisms are prompted with great potentiality. These un-traditional recognition domains or proteins are under revealing with increasing attention. 

### 5.1. Novel DM9 Domain Typically Recognizing Mannose

Recently, a novel PRR with two DM9 domains was identified from oyster *C. gigas* (*Cg*DM9CP-1) by two laboratories. DM9 domain was originally identified in *Drosophila melanogaster* with no defined functions. The DM9 containing protein (*Cg*DM9CP-1) was significantly enriched from oyster hemolymph by affinity chromatography through a mannose-conjugated cellulose column. *Cg*DM9CP-1 exhibited high binding specificity and avidity toward d-mannose residue [114]. It served as a PRR with a broad range of recognition spectrum to various PAMPs, including LPS, PGN, MAN, and β-1,3-glucan in a d-mannose-dependent manner, as well as bacteria and fungi. The crystal structures of wild-type and loss-of-function mutant of *Cg*DM9CP-1 demonstrated that Asp22 and Lys43 were the critical residues for ligand recognition [114,115]. Moreover, *Cg*DM9CP-1 protein was found to mainly distribute on the surface of *C. gigas* hemocytes, and it could be translocated into cytoplasm and colocalized with the engulfed microbes during hemocytes phagocytosis, which collectively indicated its important role as classical PRR. It is worth noting that DM9 domain has been found to exist in various proteins from a number of species, and its existence in prokaryotic cells indicates that the universal DM9 domain is an ancient protein domain that probably evolved from prokaryotes [114]. But, the conserved functions of DM9CPs domain during evolution and their intracellular signal pathways still need further investigation. A total of 477 DM9CPs have been annotated in organisms from the Procaryotae, Fungi, Protista, and Animalia Kingdoms, but just not Plantae Kingdom. The uneven distribution of phylogenetic patterns of the DM9 domain is likely to reflect the natural selection during molecular evolution in innate immunity.

### 5.2. Caspase with Specific Binding Activity to LPS

Caspases is well-known as a family of cysteine-dependent aspartate-directed proteases for their key roles in apoptosis and inflammatory responses. Recently, they were reported as the PRRs to play important role in immune recognition. Following an unexpected result that cytoplasmic LPS could activate caspase-11 “non-canonical inflammasome” in a TLR4-independent manner in mice [116], human caspase-4, -5, and mouse homologue caspase-11 were found to directly bind intracellular LPS by their CARD domain with high specificity and affinity. The intracellular LPS induced the oligomerization and activation of caspase-4/5/11, leading to pyroptosis [117]. The recognition character of caspase to LPS was favored in lower invertebrate oyster *C*. *gigas* sooner after the first publication. The oyster *Cg*Caspase-3 was confirmed to have the specific binding activity to LPS, but not to LTA, mannan, and β-1,3-glucan by ELISA-based LPS binding assay and surface plasmon resonance (SPR) analysis. The in vivo interaction of *Cg*Caspase-3 with LPS specifically inhibited the cell apoptosis, just opposite to pyroptosis promoting effect of LPS-caspase binding in mammals [118]. Interestingly, the LPS binding mechanism of *Cg*Caspase-3 was dependent on CASc domain, which was totally different from mammalian caspase-4/5/11 relying on the CARD domain. In addition, *Cg*Caspase-1, only containing one CASc domain, also displayed direct LPS binding activity, which was similar to *Cg*Caspase-3 [119]. The great evolutionary difference between mammalian and molluscan caspases encourages us to suspect that molluscs might evolve sophisticated strategies in both LPS binding mechanism and the following immune effect. It seems that the CARD-dependent LPS binding activity of mammalian caspase mainly promotes pyroptosis, while CASc-dependent LPS binding activity of molluscs caspase inhibits apoptosis. But, the detailed mechanisms and the functional differentiation among all the caspases harboring CARD or CAS domain still need further investigation.

### 5.3. IL-17 with Lectin and Bactericidal Activity

In mammals, cytokines are the major regulators of the host immune response by the interaction with specific receptors. An unexpected carbohydrate-binding (lectin) property of cytokines was discovered in the study of cytokine-receptor interaction. Some cytokines could recognize and bind to the specific glycosylation sites of their individual receptors and induce immune modulation [120,121]. For example, recombinant human interleukins IL-1α, IL-1β, IL-4, IL-6, and IL-7 exhibited different and specific calcium-independent carbohydrate-binding properties and such activities were essential for providing specific signaling systems [122,123]. The carbohydrate-recognition domains (CRDs) localized at the opposite of the receptor-binding domain endow these interleukin molecules with bi-functional regulation property [120]. However, opposite to this kind of endogenous carbohydrate binding activity, one ancient invertebrate interleukin displayed heterogenous PAMPs binding activity. The recombinant IL17-5 from oyster *C. gigas* (r*Cg*IL17-5) was proved to directly bind to PGN, LPS, poly (I:C), and β-1,3-glucan, with the highest affinity to PGN, which had never been reported in vertebrate interleukins [124]. The mRNA expression level of r*Cg*IL17-5 was strongly up-regulated after PGN stimulation, suggesting that its involvement in the recognition of diverse pathogens as a PRR and preference to recognize Gram-positive bacteria [124]. Another lectin like cytokine in earthworm *Eisenia foetida*, the coelomic cytolytic factor (CCF), displayed both lectin activity to bind LPS, PGN, and GLU, as well as TNF-like lytic activity [125]. The lectin-like activity/domain of IL and CCF may represent an essential recognition mechanism that has been functionally conserved during the innate immune response of invertebrates and vertebrates [126]. Moreover, r*Cg*IL17-5 could significantly inhibit the growth of both Gram-positive bacteria *M. luteus* and Gram-negative bacteria *E. coli*, indicating its direct anti-bacteria effect by inhibiting their proliferation [124]. The endogenous carbohydrate binding activity of vertebrate cytokine and the heterogenous PAMPs binding activity of invertebrate cytokine were suspected to be a result of functional differentiation during evolution [124].

### 5.4. Metabolic Enzymes with New Role of Carbohydrate Binding

Metabolic enzymes have long been known accustomedly for their function of catalyzing substrate to produce new products. Recently, the unexpected non-metabolic functions of metabolic enzymes were observed in the context of inflammation in both vertebrates and invertebrates. For example, the metabolic enzyme hexokinase could unexpectedly act as a pattern recognition receptor to recognize bacterial peptidoglycan and trigger the activation of inflammasome in mice [127,128]. Similarly, a phosphoenolpyruvate carboxykinase (*Cg*PEPCK) in oyster *C*. *gigas*, which is well known as a key enzyme that is involved in the metabolic pathway of gluconeogenesis, also displays the LPS and PGN binding activities [129]. Besides, arginine kinase, a conserved essential phosphagen kinase (PK) in ATP buffering systems, has also been identified (designated *Cg*AK) from the hemolymph of *C. gigas* by LPS affinity chromatography. *Cg*AK could directly bind to LPS in a concentration-dependent manner. The interaction with LPS significantly decreased the ATP hydrolytic activity of *Cg*AK and this in turn led to the accumulation of ATP in vitro, which is of great importance for the detection of pathogens and the induction of host immune responses [130]. These new progresses have subverted our traditional awareness of metabolic enzymes and inferred that their overlooked direct immune functions are evolutionarily conserved in both vertebrates and invertebrates. The latest progress in invertebrates could be also referenced for new therapeutic strategies development by exploiting these non-canonical features of metabolic machinery, modulating their contribution to the immune response without impacting their basal metabolic functions [128].

## 6. Conclusions

Recognition is the fundamental step for the interaction of hosts–pathogens. Many glycans show remarkably discontinuous distribution across various microbes, and certain lineage-specific glycans have become important signals for nonself recognition of hosts by a toolkit of innate recognizing molecules [131]. Coincidentally, the recognition domains in multicellular organisms, especially in invertebrates, also show significantly species-specific expansion in genome [17]. A lot of members of the expanded PRR families have been confirmed to have versatile functions in immune response, and their functional cooperation and differentiation might provide a subtle discrimination of self from nonself. It could be inferred that there might be a correlationship between the lineage-specific glycan of pathogens and species-specific expansion of innate recognition domains in host. However, the detailed mechanism about the species-specific interaction between versatile innate recognition receptors with some common domains and microbes need much more researches. In molluscs, the great biodiversity of species provide plenty of solutions for pathogen recognition, and the presence of open circulatory system and ancient specialized hemocytes offer effective immunological surveillance. A systemic comparison of the heterogeneous PRRs might be needed for better understanding their functional differentiation. Meanwhile, with the rapid development of multi-omics biotechnology, the comprehensive and systematic understanding about the carbohydrate recognition during host-microbes interaction and the other processes might be revealed by big data analysis of genome information in hosts and great glycomic data in pathogens.

## Figures and Tables

**Table 1:** The main component of common pathogen-associated molecular patterns (PAMPs).

PAMPs	Pathogens	Carbohydrates	Proteins	Nucleic Acids	Lipids
LPS	G−	+			+
PGN	G+	+	+		
LTA	G+	+	+		
Flagellin	G+/G−	+			
Lipoprotein	G+/G−	+	+		
MAN	fungi	+			
Poly I:C	virus			+	
CpG	virus			+	

Lipopolysaccharide, LPS; peptidoglycan, PGN; mannan, MAN; lipoteichoic acid, LTA.

**Table 2:** The main recognition domains of various pattern recognition receptors (PRRs).

PRRs	Ig	LRR	Lectin	C1q	SR
FREP	+				
TLR		+			
CTL			+		
C1qDC				+	
SR					+

Fibrinogen-related protein, FREP; Toll like receptor, TLR; C-type lectin receptor, CTL; C1q domain containing protein, C1qDC; scavenger receptor, SR.
